# Development of a droplet digital polymerase chain reaction tool for the detection of *Toxoplasma gondii* in meat samples

**DOI:** 10.1007/s00436-022-07477-9

**Published:** 2022-03-01

**Authors:** Andrea Mancusi, Angela Giordano, Antonio Bosco, Santa Girardi, Yolande T. R. Proroga, Luigi Morena, Renato Pinto, Paolo Sarnelli, Giuseppe Cringoli, Laura Rinaldi, Federico Capuano, Maria Paola Maurelli

**Affiliations:** 1 Istituto Zooprofilattico Sperimentale del Mezzogiorno, Portici, NA) Italy; 2 Unit of Parasitology and Parasitic Diseases, Department of Veterinary Medicine and Animal Production, University of Naples Federico II, CREMOPAR, Naples, Italy; 3 Centro Di Riferimento Regionale Sanità Animale (CReSan), Salerno, Italy; 4 UOD Prevenzione E Sanità Pubblica Veterinaria Regione Campania, Naples, Italy

**Keywords:** *Toxoplasma gondii*, Toxoplasmosis, Droplet digital polymerase chain reaction (ddPCR), Quantitative PCR

## Abstract

Toxoplasmosis is a zoonotic disease caused by the protozoan parasite *Toxoplasma gondii*. Infection in humans has usually been related to the consumption of raw, undercooked or cured meat. The aim of this study was to develop a droplet digital polymerase chain reaction (ddPCR)-based assay for the detection and quantification of *T. gondii* in meat samples. To optimize the ddPCR, *T.gondii* reference DNA aliquots at five known concentrations: 8000 cg/µl, 800 cg/µl, 80 cg/µl, 8 cg/µl were used. Moreover, results obtained by ddPCR and quantitative PCR (qPCR) were compared using 80 known samples (40 positive and 40 negative), as well as 171 unknown diaphragm tissue samples collected at slaughterhouses. The ddPCR showed a sensitivity of 97.5% and a specificity of 100%, with a detection limit of 8 genomic copy/µl of *T. gondii*. A nearly perfect agreement (*κ* = 0.85) was found between results obtained by ddPCR and qPCR for both positive and negative known samples analysed. On the 171 diaphragm tissue samples from field, 7.6% resulted positive by ddPCR and only 1.2% by qPCR. Therefore, this innovative method could be very useful for the detection of *T. gondii* in meat samples, aiming to prevent human infections.

## Introduction

Toxoplasmosis, caused by the intracellular protozoan *Toxoplasma gondii*, is one of the most common parasitic infection in animals and humans worldwide (Almeira and Dubey, 2021). Domestic and wild felids are the definitive hosts of this parasite and shed in the environment the oocysts that become infective after sporulation, representing a risk for other definitive or intermediate hosts (potentially all the warm-blooded animals, including birds, marine mammals and humans) (Dubey et al., 2020).

As reported by FAO/WHO (2014), toxoplasmosis is considered one of the most important food-, water- and soil-borne diseases. It is estimated that approximately two billion of people are infected with *T. gondii* (Almeria and Dubey, 2021). In a report on foodborne diseases by European Food Safety Authority (EFSA), *T. gondii* was ranked third in Europe (EFSA, 2018).

The main routes of infection are ingestion of: (i) food or water contaminated with sporulated oocysts (e.g. vegetables, fruit and molluscan shellfish); (ii) uncooked or undercooked meat containing tissue cysts (Ghozzi et al., 2017; Caradonna et al., 2017; EFSA, 2018). Moreover, tachyzoites excreted in milk could be a source of infection. Indeed, outbreaks of toxoplasmosis associated with consumption of unpasteurized goats’ milk have been reported (FAO/WHO, 2014; EFSA, 2018; Almeria and Dubey, 2021). Tachyzoites can also be transmitted vertically from mother to foetus or via organ transplants (Smith et al., 2021).

Although in immunocompetent people toxoplasmosis can usually be asymptomatic or mild symptomatic, this parasite can cause severe consequences in immunocompromised hosts, i.e. ophthalmitis, encephalitis, pneumonitis and myocarditis which can also be fatal (Smith et al., 2021). Moreover, an association between *T. gondii* infection and neuropsychiatric disorders, as well as personality changes have been reported in several studies (Chaudhury and Ramana, 2019; Almeria and Dubey, 2021). If women become infected during the pregnancy period, miscarriage, stillbirth or congenital defects may occur that can be immediately visible in the newborn or may develop during their lifetime (e.g. neurological problems, mental retardation, deafness and/or ocular lesions) (Almeria and Dubey, 2021).

In animals, symptoms may vary depending on the species. Tachyzoites can be transmitted vertically especially in sheep and goats. However, as in humans, abortion, stillbirth and neonatal death have been reported, mostly in small ruminants, causing economic losses for farmers (Almeria and Dubey, 2021). No confirmed clinical toxoplasmosis reports have been reported in cattle (Lindsay and Dubey, 2020).

Several direct and indirect techniques have been used to detect *T. gondii* in intermediate hosts and in food products. Among direct techniques, cat and mouse bioassays are the reference methods for assessing viability of the parasite. However, these tests are not easy to use, considering the long time to obtain results and ethical issues, as well as costs (Guo et al., 2015; EFSA 2018; Almeria and Dubey, 2021). Cell cultures can be a valid alternative to bioassays, but they are limited used and protocols are described mainly for fluid samples, while meat homogenated samples gave variable results (Warnekulasuriya et al., 1998; EFSA 2018, Opsteegh et al., 2020; Almeria and Dubey, 2021).

Home-made or commercially available indirect serological tests, e.g. immunofluorescence assay (IFAT), enzyme-linked immunosorbent assay (ELISA), latex agglutination tests (LAT), modified agglutination tests (MAT) and hemagglutination assay (HA), are most commonly used to identify *T.gondii* positive farms and individual animals, especially for epidemiological surveys on a large number of samples (EFSA, 2018; Almeria and Dubey, 2021). Meat juice has been proven to be an excellent matrix for serological studies on *T. gondii* in different host species, i.e. sheep, pigs, wild boars, cattle and chickens (Meemken and Blaha, 2011; Basso et al., 2013; Meemken et al., 2014; Bacci et al., 2015; Vismarra et al., 2016, 2017; Slany et al., 2016; Felin et al., 2017; Schares et al., 2018; Olsen et al., 2020; Gazzonis et al., 2020).

Moreover, new serological tools have been developed for the detection of *T. gondii* in chickens, using Luminex technology (Fabian et al., 2020), a luciferase-linked antibody capture assay (LACA) (Duong et al., 2020) or microarrays to detect antibodies in meat juice and serum (Loreck et al., 2020).

The molecular biology methods are the most widely used direct techniques. Several PCR protocols have been described: end-point, nested PCR and real-time PCR (qPCR) to amplify the B1 gene or the 529 bp repeat element that are the most used targets (Reischl et al., 2003; EFSA, 2018; Almeria and Dubey, 2021). To increase the sensitivity of PCR-based diagnostic methods, a magnetic-capture-(MC-) real-time PCR for detection of *T. gondii* in meat was developed by Opsteegh et al. (2010), applied to sheep and chicken meat by Schares et al. (2018) and improved by Gisbert Algaba et al. (2017) for diagnosis in pork meat. Moreover, some loop-mediated isothermal amplification (LAMP) protocols have also been developed for the early detection of *T. gondii* as alternative method to the above-mentioned PCR methods, to increase the sensitivity of available diagnostic techniques (Zhang et al., 2009; Lin et al., 2012; Qu et al., 2013; Zhuo et al., 2015). A commercialized LAMP assay is available for the diagnosis of toxoplasmosis in humans (Varlet-Marie et al., 2018). Moreover, an adaptation of the LAMP technique has been combined with a lateral flow dipstick chromatographic detection system for a rapid visualization of results to detect oocysts in vegetable products (Lalle et al., 2018).

However, the development of new more sensitive and specific diagnostic tools is still ongoing. The droplet digital polymerase chain reaction (ddPCR) is a new PCR method that provides absolute and direct quantification of target DNA, without the need of a standard curve like the qPCR, with a higher sensitivity than other PCR methods (Hindson et al., 2011, 2013).

The ddPCR has been successfully used for detection of different parasites, e.g. *Cryptosporidium* in different animal hosts and in humans, *Echinococcus multilocularis* in meadow voles and deer mice, *Dirofilaria immitis* in dogs, *Cytauxzoon felis* in cats, gastrointestinal nematodes and *Trichuris* spp., in sheep and cattle, *Eimeria* spp. *Ascaridia galli* and *Heterakis gallinarum* in chickens, *Babesia microti*, *Babesia duncani*, *Plasmodium* spp., *Strongyloides stercoralis* and *Schistosoma japonicum* in humans (Yang et al., 2014; Wilson et al., 2015; Weerakoon et al., 2016; Srisutham et al., 2017; Baltrusis et al., 2019; Mahendran et al., 2020; Yu et al., 2020; Shang Kuan and Pichard, 2020; Kao et al., 2021; Tarbiat et al., 2021; Snyder et al., 2021; Iamrod et al., 2021; Massolo et al., 2021).

The aim of this paper is to develop and validate a new ddPCR assay for detection and quantification of *T. gondii* DNA in meat of intermediate hosts.

## Materials and methods

### Preparation of DNA samples

To optimize the ddPCR, specific reference strains were obtained from the American Type Culture Collection (ATCC). The stock solution of *Toxoplasma gondii* ATCC 50174D contained ~ 2 × 10^5^ cg/µl genome equivalent.

Twenty-five grams of 80 negative samples of minced meat from cattle was homogenized by a stomacher, then total DNA was extracted, using a QIAamp DNA Mini kit (Qiagen, Hilden, Germany), according to the manufacturers’ instructions. The negativity of the samples used was evaluated using the qPCR protocol suggested by the National Reference Centre for toxoplasmosis (Palermo, Italy).

DNA concentration was determined by a Biophotometer (Eppendorf, Hamburg, Germany), then samples were diluted to be analysed by ddPCR at five concentration levels: 8000 cg/µl, 800 cg/µl, 80 cg/µl, 8 cg/µl, to evaluate the limit of detection at 95% of probability (LOD_95_).

Sensitivity was determined using 40 DNA samples extracted from negative minced meat experimentally contaminated with DNA from the *T. gondii* ATCC. Ten replicates were prepared for each concentration. Forty samples inoculated only with sterile water were used as negative controls.

### Optimization of the ddPCR

Primers and probe (Applied Biosystems, Foster City, CA, USA) used to amplify the region Toxo-529 bp repeat element of the parasite were: forward AF1 CACAGAAGGGACAGAAGT; reverse AF2 TCGCCTTCATCTACAGTC; probe FAM CTCTCCTCCAAGACGGCTGG BHQ (Pepe et al., 2021). Their specificity was evaluated in silico using the NCBI nucleotide BLAST tool and by ddPCR using positive samples for another abortive agent, *Neospora caninum*.

The annealing temperature for *T. gondii* was optimized using a thermal gradient (specifically, 56, 56.4, 57.2, 58.4, 59.8, 61, 61.7, and 62 °C temperatures were tested) in a CFX96 (Bio-Rad, Hercules, CA, USA), before developing the ddPCR.

The ddPCR was performed using the QX200 system (Bio-Rad, Hercules, CA, USA). The mastermix was prepared in a total volume of 20 μl, mixing 10 μl of ddPCR Supermix for probes (Bio-Rad, Hercules, CA, USA), 0.5 µM forward primer, 0.5 µM reverse primer, 0.25 µM probe and 35–50 ng for reaction of DNA (Pepe et al., 2021). The reaction mixture was transferred to the DG8 cartridge (Bio-Rad, Hercules, CA, USA). A volume of 70 μl of droplet generation oil was added into the oil well and droplets were formed in the droplet generator (Bio-Rad, Hercules, CA, USA). Then, 40 μl of droplet-partitioned samples were transferred to a 96-well plate and sealed with the specific device. The PCR amplification was carried out on a CFX96 instrument (Bio-Rad, Hercules, CA, USA) with the following thermal profile: 96 °C for 10 min followed by 45 cycles at 98 °C for 30 s, 58.5 °C for 1 min and a final stage at 98 °C for 10 min. After thermal cycling, the 96-well plate was read in the QX200 Droplet Reader, based on positive droplets, according to the Poisson distribution. QuantaSoft software was used to count the PCR-positive and PCR-negative droplets to provide absolute quantification of the target DNA. The quantification measurements of each target were expressed as the number of genomic copies per 1 µl of reaction.

Intra-laboratory repeatability validation was performed by different operators to verify the robustness of the established ddPCR method, calculating the coefficient of variation (CV%) between the assays. Serial dilutions of *T. gondii* ATCC 50174D genomic DNA from 2 × 10^5^ to 2 gc/µl were analysed.

To evaluate the performance of the ddPCR, the 80 prepared samples (40 negative and 40 positive) were also analysed by qPCR, according to the protocol described in Pepe et al. (2021).

### Validation of the developed ddPCR

Overall, 171 diaphragmatic tissue samples (60 cattle, 40 buffaloes, 34 sheep and 37 pigs) collected in slaughterhouses were used for ddPCR validation. An aliquot of 25 g of each sample was weighted and subjected to DNA extraction with the QIAamp DNA Mini commercial kit (Qiagen, Hilden, Germany).

DNA samples were analysed by qPCR (Pepe et al., 2021) and ddPCR (as described above) to compare the results obtained.

The Standards for Reporting of Diagnostic Accuracy Studies (STARD) checklist (https://www.equator-network.org/reporting-guidelines/stard/) was used to report results on performances of techniques (Cohen et al., 2016).

### Statistical analysis

The intra-assay CVs for each dilution level (8000, 800, 80 and 8 cg/ml) and overall were calculated by dividing the standard deviation/the arithmetic mean concentration value [(CV% = standard deviation [SD]/mean value for each level) × 100].

Sensitivity, specificity, negative and positive predictive values (NPV and PPV) were calculated for ddPCR, considering the Rt-PCR as gold standard. The agreement between qPCR and ddPCR was calculated using Cohen’s *κ* statistic (Thrusfield, 2007).

The *κ* measure was interpreted as follows: 0, no agreement; 0.01–0.20, poor agreement; 0.21–0.40, fair agreement; 0.41–0.60, moderate agreement; 0.61–0.80, substantial agreement; and 0.81–1.0, nearly perfect agreement (Thrusfield, 2007).

The 95% confidence interval (95% *CI*) was calculated using the free online software “Sample Size Calculator” (Creative Research Systems, CA, USA).

## Results

### Optimization of the ddPCR

The optimal annealing temperature for ddPCR was 58.5 °C. This temperature was chosen considering the best differentiation of fluorescence between positive and negative samples and avoiding unspecific amplification. No cross-reactions with other parasites, as *N. caninum* were found in silico and ddPCR assays.

**Fig. 1:**
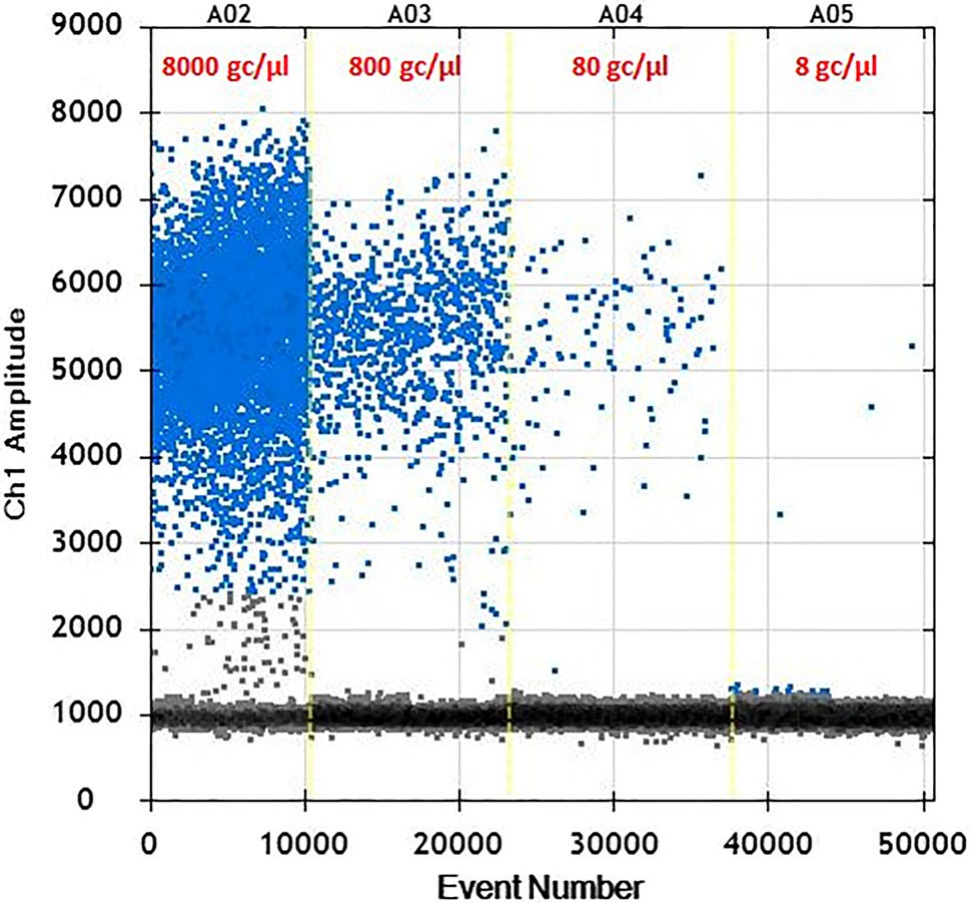
Amplification plot obtained to evaluate the LOD_95_ in ddPCR

The LoD_95_ obtained for ddPCR was 8 gc/µl in ddPCR (Fig. 1). The sample was considered positive if showed ≥ two droplets. No positive droplets were detected in the samples used as negative controls. The number of droplets generated for reaction ranged from 8985 to 13,940, with an average of 11,384 droplets. Reactions with more than 8000 accepted droplets for well were used for analysis. The ddPCR data revealed good separation between negative and positive droplets with few interface droplets supporting a high primer specificity and reaction efficiency. At high concentrations (> 10,000 gc/µl), droplets were positively saturated, making the Poisson algorithm invalid and resulting in a relative narrower dynamic range than qPCR.

The sensitivity of ddPCR was 97.5% (95% *CI* = 85.3–99.9) and specificity 100%. The performance of ddPCR is reported in Table 1. An overall *CV*% = 9.4 was calculated for all the ddPCR positive replicates (8000 gc/µl *CV*% = 3.9, 7800 cg/µl *CV*% = 2.7, 80 cg/µl *CV*% = 7.4, 8 cg/µl *CV*% = 23.6). No significant intra-laboratory variation in results was reported (*CV*% < 0.1).

**Table 1:** Performances of ddPCR for *T. gondii* detection and quantification

Performance	ddPCR (%; 95% *CI*)
Sensitivity	97.5; 85.3–99.9
Specificity	100; 89.1–99.8
NPV	97.6; 85.6–99.9
PPV	100; 89.1–99.8

A nearly perfect agreement (*κ* = 0.85; *p* < 0.0001) was found between results obtained by ddPCR and qPCR for positive and negative samples analysed in the development phase. In Table 2, there are reported concentration values obtained by ddPCR and Ct values obtained by qPCR for each level tested.

**Table 2:** Concentration values (gc/µl) obtained for each dilution level (8000 gc/µl, 800 gc/µl, 80 gc/µl and 8 gc/µl) by ddPCR and corresponding Ct values obtained by real-time PCR

8000 gc/µl		800 gc/µl		80 gc/µl		8 gc/µl	
ddPCR (gc/µl)	Real-time PCR (ct)	ddPCR (gc/µl)	Real-time PCR (ct)	ddPCR (gc/µl)	Real-time PCR (ct)	ddPCR (gc/µl)	Real-time PCR (ct)
850	23.41	92	27.12	8.40	30.93	1.60	N/A
918	23.63	87	26.76	8.50	31.34	1.30	N/A
974	23.53	81	26.76	6.90	30.81	1.60	36.92
873	23.55	86	27.04	9.40	31.47	1.00	36.55
927	23.43	79	27.10	8.70	30.89	1.60	N/A
977	23.57	79	26.62	11.0	31.42	1.90	N/A
929	23.39	87	26.90	10.8	30.98	0.61	N/A
1019	23.56	87	26.95	9.30	31.18	0.44	36.25
1076	23.70	88	26.90	10.4	30.56	1.10	N/A
979	23.55	85	26.90	8.70	30.79	0.84	35.68

### Validation of ddPCR

Of the 171 samples examined, the qPCR reference method detected *T. gondii* in only two samples (1.2%; 95% *CI* = 0.2–4.6) while ddPCR detected 13 positive samples (7.6%; 95% *CI* = 4.3–12.9). The positive samples not detected by qPCR showed concentrations ranging from 0.3 to 17.1 gc/µl. None of the samples examined, showed an inhibitory effect on PCR, as evidenced by the results of the Internal Amplification Control (IAC).

## Discussion

The European Food Safety Authority (EFSA) has suggested that meat-borne transmission accounts for around 60% of human *T. gondii* infections (EFSA, 2018; Almeira and Dubey, 2021). The main sources of contaminated meat are pork and mutton (Almeira and Dubey, 2021). Although different PCR protocols (i.e. PCR end-point, nested, semi-nested and qPCR) have been developed to detect *T. gondii* DNA in meat, many published studies have shown that this molecular approach is not very sensitive, due to the inhomogeneous distribution of *T. gondii* tissue cysts and the small size of the sample used for the analysis (Opsheegh et al., 2010; EFSA, 2018).

For these reasons, more innovative and alternative methods have been developed to increase sensitivity, e.g. the magnetic capture-PCR (mcPCR) and LAMP (Herrmann et al., 2012; Dubey et al., 2021).

In this study, promising results were obtained by ddPCR (7.6% of positive samples to *T. gondii vs* 1.2% obtained by qPCR). This innovative approach, like qPCR, does not require sequencing of amplified products, because fluorescent-labelled, target-specific probes are used to recognize a desired target.

The main advantage of the ddPCR technique is based on water–oil droplet emulsion technology to distribute the sample solution into ~ 20,000 partitions, increasing the sensitivity, accuracy and precision to detect and quantify also small quantity of the target to amplify (Kao et al., 2021). Moreover, no standard curve is necessary to quantify DNA, indeed droplets contain more target copies and the absolute count can be calculated using Poisson statistics, permitting to evaluate also small differences in target DNA copy numbers among samples (Hindson et al., 2011). Therefore, the ddPCR is more reproducible than qPCR, indeed it is less operator or laboratory dependent, because there is a lower possibility of errors related to pipetting steps to prepare serial dilutions or misinterpretation due to incorrect preparation of standard curve, so a reliable comparison of quantification of target DNA copies can be performed in different laboratories (Huggett et al., 2008). Moreover, the ddPCR is relatively insensitive to potential PCR inhibitors, such as providing lower variation between replicates; therefore, ddPCR is more repeatable than qPCR (Campomenosi et al., 2016).

Further studies will be needed to confirm our preliminary results and to analyse other matrices. Indeed, a limitation of this study is the small number of the positive samples found during the validation phase under “field conditions”.

However, this innovative approach could be very useful for a rapid detection of small amounts of *T. gondii* in meat, as well as in other food matrices, e.g. milk, cheese, vegetables and molluscs, so to perform valid control strategies, aimed to reduce the risks of toxoplasmosis infection in animals and humans, according to the One Health approach.
